# Isocorydine Exerts Anticancer Activity by Disrupting the Energy Metabolism and Filamentous Actin Structures of Oral Squamous Carcinoma Cells

**DOI:** 10.3390/cimb46010042

**Published:** 2024-01-09

**Authors:** Qiaozhen Zhou, Qianqian Zhang, Lingzi Liao, Qian Li, Huidan Qu, Xinyu Wang, Ying Zhou, Guangzeng Zhang, Mingliang Sun, Kailiang Zhang, Baoping Zhang

**Affiliations:** 1 Department (Hospital) of Stomatology, Lanzhou University, Lanzhou 730000, China; lzu_zhouqz@lzu.edu.cn (Q.Z.); zhangqq2021@lzu.edu.cn (Q.Z.); liaolz20@lzu.edu.cn (L.L.); 220220929210@lzu.edu.cn (Q.L.); quhd21@lzu.edu.cn (H.Q.); wangxinyu21@lzu.edu.cn (X.W.); zhouying21@lzu.edu.cn (Y.Z.); 220220929721@lzu.edu.cn (G.Z.); 220220929441@lzu.edu.cn (M.S.); 2 Key Laboratory of Dental Maxillofacial Reconstruction and Biological Intelligence Manufacturing, Lanzhou University, Lanzhou 730000, China

**Keywords:** isocorydine, oral squamous cell carcinoma, anticancer activity, energy metabolism

## Abstract

Isocorydine (ICD) exhibits strong antitumor effects on numerous human cell lines. However, the anticancer activity of ICD against oral squamous cell carcinoma (OSCC) has not been reported. The anticancer activity, migration and invasion ability, and changes in the cytoskeleton morphology and mechanical properties of ICD in OSCC were determined. Changes in the contents of reactive oxygen species (ROS), the mitochondrial membrane potential (MMP), ATP, and mitochondrial respiratory chain complex enzymes Ⅰ–Ⅳ in cancer cells were studied. ICD significantly inhibited the proliferation of oral tongue squamous cells (Cal-27), with an IC50 of 0.61 mM after 24 h of treatment. The invasion, migration, and adhesion of cancer cells were decreased, and cytoskeletal actin was deformed and depolymerized. In comparison to an untreated group, the activities of mitochondrial respiratory chain complex enzymes I-IV were significantly decreased by 50.72%, 27.39%, 77.27%, and 73.89%, respectively. The ROS production increased, the MMP decreased by 43.65%, and the ATP content decreased to 17.1 ± 0.001 (mmol/mL); ultimately, the apoptosis rate of cancer cells increased up to 10.57% after 24 h of action. These findings suggest that ICD exerted an obvious anticancer activity against OSCC and may inhibit Cal-27 proliferation and growth by causing mitochondrial dysfunction and interrupting cellular energy.

## 1. Introduction

Oral squamous cell carcinoma (OSCC) is one of the most common malignancies of the head and neck, characterized by a high recurrence rate and drug resistance [1]. Despite recent advances in techniques for the diagnosis and treatment of OSCC, the 5-year survival rate of OSCC patients has stagnated at 50% [2,3]. Therefore, new drugs are being developed and researched to provide additional therapeutic options.

At present, tumor therapies involve surgery, radiotherapy, chemotherapy, molecular targeted therapy, and multi-method combinations. However, various herbs have been reported to exert antitumor activity, such as Isorhapontigenin (ISO) [4], Chamaejasmine [5], Lycium barbarum polysaccharide (LBP) [6], and Oridonin [7]. Many of them have been used in combination with other chemotherapeutic agents in the treatment of various cancers, effectively reducing side effects and enhancing the efficacy of the drugs. Furthermore, herbs or their monomer extracts have also been reported to enhance the radiosensitivity of cancer cells due to their low toxicity, mild action, and strong antitumor activity [8]. In recent years, the antitumor efficacy of various herbs in OSCC, such as Neferine (NEF) [9], Danshensu [10], Deoxypodophyllotoxin [11], and Solanum lyratum (SLEC) has been described [12]. These herbs effectively inhibit oral squamous carcinoma cell proliferation, induce apoptosis, and reduce resistance to other chemotherapeutic agents; therefore, they enhance antitumor efficacy and can be used alone or in combination with other chemotherapy agents [13,14]. Considering the high efficacy of herbal medicines in the treatment of OSCC, efficient and safe antitumor drugs can be developed for the clinical treatment of OSCC.

Isocorydine (ICD) is an aporphine alkaloid that is found in 20 families and more than 100 plant genera, exhibiting excellent biological activity and therapeutic potential [15,16]. ICD has antiarrhythmic [17], antibacterial [18], antiulcer [19], antitumor [20], antiplasmodium, and antioxidant properties [21]. Furthermore, it has been reported to inhibit the proliferation of various tumor cell lines, such as the cell lines of liver cancer, gastric cancer, lung cancer, pancreatic cancer, and cervical cancer [15,22]. Previous studies reported that ICD and its derivatives can inhibit cancer cell proliferation through many pathways, such as the induction of G2/M cell cycle arrest and apoptosis [23], a reduction in insulin-like growth factor 2 mRNA binding protein 3 (IGF2BP3) expression [24] the downregulation of ITGA1 expression [25], and the regulation of apoptotic process PDCD4 expression [26]. Moreover, ICD can be combined with first-line anticancer drugs such as gemcitabine [22], doxorubicin (DOX) [27], and cisplatin to enhance anti-cancer sensitivity and reduce drug resistance [26]. Nevertheless, the efficacy of ICD in anti-OSCC treatment has not been explored.

Tumor cells undergo altered energy metabolism which mainly relies on the glycolysis pathway. This pathway is an inefficient ATP production system that generates large amounts of lactate and pyruvate, known as the “Warburg effect” or “aerobic glycolysis”. [28]. In particular, mitochondria play a dominant role in mediating apoptosis and have been suggested as a new pharmacological target for cancer therapy [29,30]. Mitochondrial dysfunction can interrupt the energy supply and activate mitochondria-mediated apoptotic pathways [31]. Anticancer drugs have been found to cause oxidative stress in cancer cells, characterized by increased reactive oxygen species (ROS) activity, decreased ATP levels, and the depolarization of the mitochondrial membrane potential (MMP). Mitochondria-targeted enhanced radiotherapy depolarizes the MMP, releases cytochrome c, and disrupts the activity of mitochondrial respiratory chain complex enzymes I-IV, decreasing respiratory efficiency and cellular oxygen consumption, ultimately leading to programmed cell death [32,33,34].

Therefore, this study explored whether ICD can be applied to the treatment of OSCC and improve the anticancer effect. In this study, ICD was hypothesized to inhibit the proliferation of Cal-27 and the related cell energy metabolism and cytoskeletal changes, providing a new idea for drug therapy for OSCC.

## 2. Results

### 2.1. ICD Inhibited Oral Squamous Cell Carcinoma Growth

**Figure 1:**
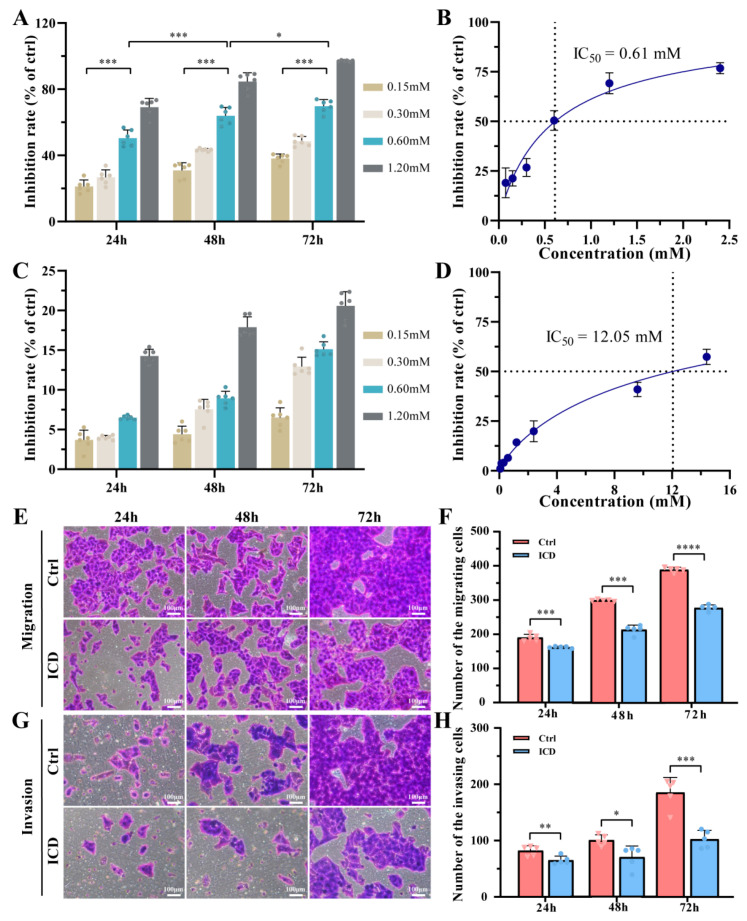
The inhibitory effect of ICD on the proliferation of Cal-27 (**A**) and HGF (**C**) cells was detected using the CCK-8 method. Half-mortality curves of ICD against Cal-27 (**B**) and HGF (**D**) cells. The anti-migration effect of ICD (**E**,**F**). The anti-invasion effect of ICD (**G**,**H**). * *p* < 0.05; ** *p* < 0.01; *** *p* < 0.001; **** *p* < 0.0001. The ICD concentration was 0.60 mM.

A significant difference in the inhibitory effect of ICD on Cal-27 cells was observed after 24 h, as displayed in Figure 1A. The inhibition rate of Cal-27 cell proliferation reached 50.47% in the 0.60 mM ICD group, while it was only 6.49% after HGF cells were treated at the same concentration (Figure 1C). As shown in the 48 h and 72 h treatment groups, ICD inhibited Cal-27 cell proliferation in a dose-dependent and time-dependent manner. After 72 h of high-ICD-concentration treatment (2.40 mM), the proliferation inhibition rate of Cal-27 cells was over 97%, whereas it was only 31.01% for HGF cells, which indicates that at specific concentrations ICD effectively inhibits the growth of tumor cells without causing serious fatal effects for normal cells. The findings demonstrate the significant anticancer activity of certain concentrations of ICD compared to the control group, thus laying a foundation for subsequent related studies. The median inhibitory concentration (IC50) was 0.61 mM after 24 h of ICD culture (Figure 1B), and the HGF cell inhibition was 50% only when the ICD concentration was 12.05 mM (Figure 1D). Therefore, an ICD concentration of 0.60 mM was selected as the experimental concentration based on the half-fatality curve.

### 2.2. ICD Decreased the Migration and Invasion Ability of OSCC

**Figure 2:**
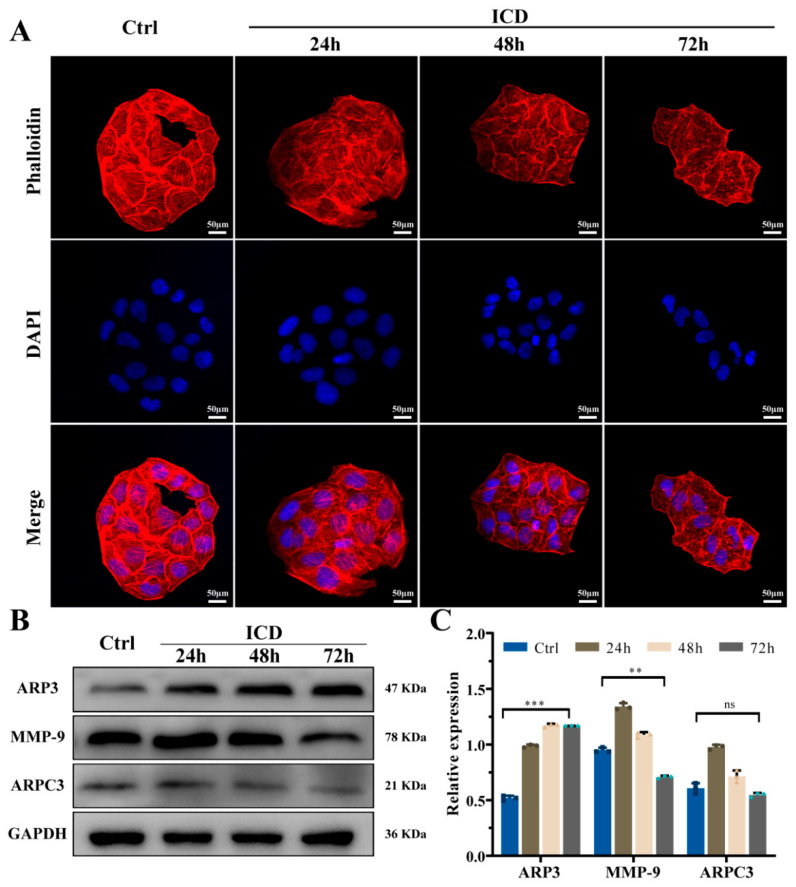
Stained cytoskeleton images after treatment with ICD for 24 h, 48 h, and 72 h (**A**). Actin-related protein expression of Cal-27 was detected via Western blotting (**B**,**C**). ** *p* < 0.01; *** *p* < 0.001; ns, non-significant. The ICD concentration was 0.60 mM.

After incubation with 0.60 mM ICD for 24 h, 48 h, and 72 h, a significant reduction in cell migration was observed at 24 h (*p* < 0.001), with a significantly larger discrepancy between the two groups after 48 h and 72 h (*p* < 0.0001), as shown in Figure 1E,F. The invasion experiment (Figure 1G,H) showed that the number of cell invasions was significantly reduced compared to the control group, (*p* < 0.01), and the difference was still statistically significant after 48 h and 72 h (*p* < 0.05). After 72 h of ICD treatment, the expression of MMP-9 was downregulated (Figure 2B,C), which also confirmed the decreased migration ability of the cells. ICD significantly reduced the migration and invasion ability of cancer cells, with more significant effects as the ICD treatment time increased.

### 2.3. Depolymerized Cytoskeleton and Reduced Cell Adhesion in Response to ICD

Cytoskeletal morphological changes are shown in Figure 2A. A narrowed cell morphology and a disordered intracellular skeleton were observed after 24 h of ICD treatment. After 48 h and 72 h, the cell morphology was significantly narrowed, the cytoskeletal actin was significantly degraded, and the cytoskeleton collapsed and the boundary was blurred. The cytoskeletons of oral squamous carcinoma cells were significantly constricted, degraded, and collapsed after exposure to ICD, an outcome which intensified with longer exposure. These results were consistent with the migration and invasion assay results.

Subunits of the actin-related protein 2/3 complex (a major component of filamentous actin), ARP3 and ARPC3, may have a role in the migration of cancer cells and the development of tumors. To further validate this result, WB experiments (Figure 2B,C) were performed which showed that ICD induced a significant increase in ARP3 expression which became more pronounced with the duration of treatment. And there was no significant change in ARPC3 expression. The results suggest that the cytoskeleton was degraded and collapsed by ICD, and actin-related protein ARP3 was released from the Arp2/3 complex, leading to increased levels.

**Figure 3:**
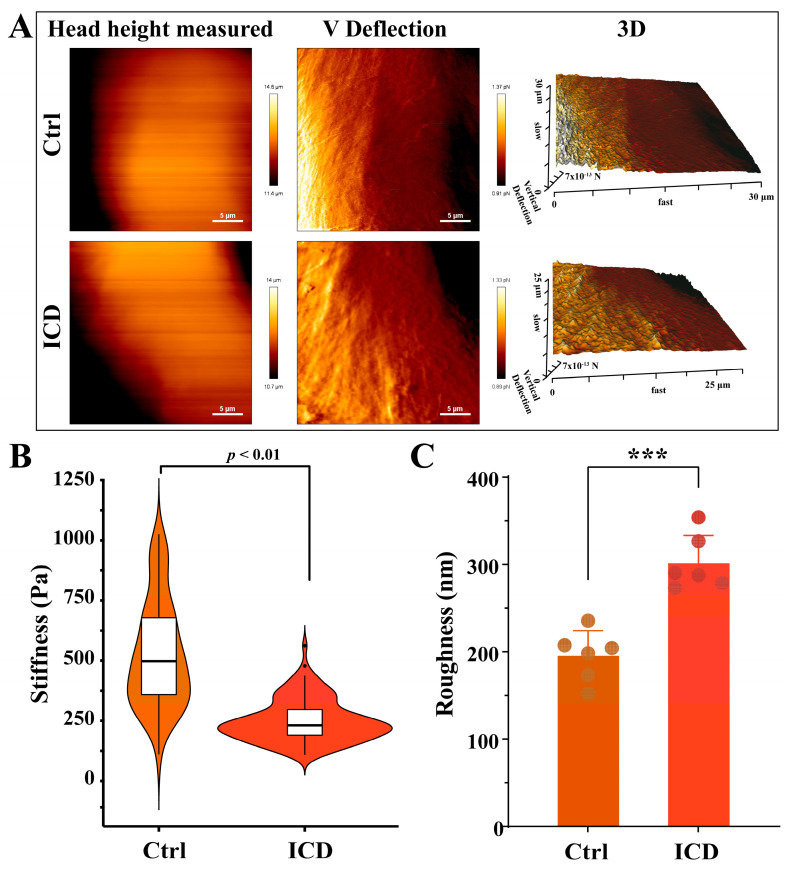
Surface morphology (**A**), stiffness (**B**), and roughness (**C**) were detected via AFM after ICD treatment for 24 h. *** *p* < 0.001. The ICD concentration was 0.60 mM.

The atomic force microscope (AFM) has been extensively developed for applications in actin structural properties. AFM results showed that the cell membrane of the control group was smooth and complete, with a plump and bulging nucleus (Figure 3A). The average roughness values of the cell surface were 301.6 nm and 195.1 nm with and without 0.60 mM ICD, respectively (Figure 3C). After exposure to ICD, the cells were constricted and deformed, and the surface of the cell membrane was rough and fluctuated greatly, showing serious damage. In addition, irregularly arranged actin filaments were observed in tumor cells of the experimental group. ICD induced the degradation and dispersion of the fine cellular skeleton structures/units, and the actin filaments were almost dissolved. Finally, an observation and analysis of cell stiffness showed a significant downward trend in the elastic modulus after ICD treatment (*p <* 0.001) (Figure 3B).

### 2.4. ICD Disrupted Cellular Energy Metabolism and Accelerated Apoptosis

**Figure 4:**
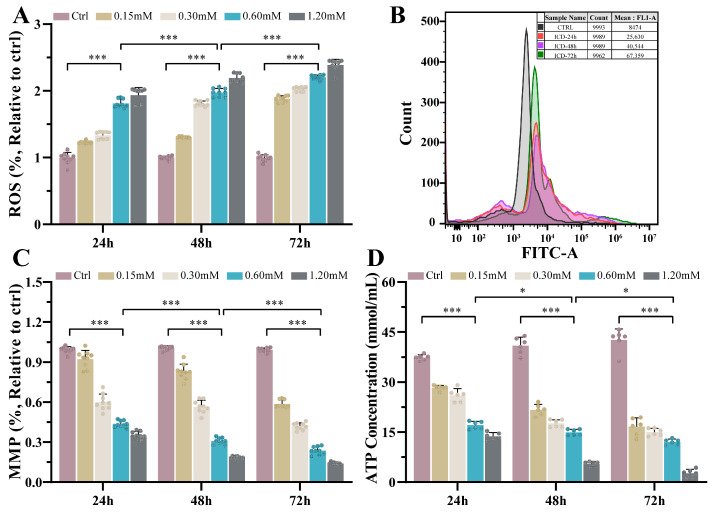
Effects of different concentrations of ICD on ROS (**A**,**B**), MMP (**C**), and ATP (**D**) in Cal-27. * *p* < 0.05; *** *p* < 0.001.

After ICD treatment with different concentrations for 24 h, 48 h, and 72 h, ROS production in oral squamous carcinoma cells was detected, as displayed in Figure 4A,B. ROS production also increased with longer exposure (*p <* 0.001). The reactive oxygen species content at 72 h of ICD action was about three times that at 24 h (Figure 4B). ICD may induce a significant increase in ROS production in oral squamous carcinoma cells and cause mitochondrial damage in cancer cells. Mitochondrial function was also correlated with changes in the mitochondrial membrane potential and intracellular ATP content. As shown in Figure 4C, the MMP significantly decreased, and cell depolarization was obvious after ICD treatment, which further decreased with a longer exposure duration and higher ICD concentration (*p* < 0.001). Furthermore, changes in the intracellular ATP content were detected (Figure 4D). Following 0.60 mM ICD treatment for 24 h, 48 h, and 72 h, a significant decrease in ATP content was observed compared with the control group (*p* < 0.001). The ATP content further decreased with longer ICD exposure (*p* < 0.05, *p* < 0.001).

Moreover, the activities of mitochondrial respiratory chain complex enzymes I–IV were tested, as shown in Table 1. ICD effectively reduced the activities of NADH dehydrogenase, succinate dehydrogenase, cytochromic reductase, and cytochromic oxidase to varying degrees. After 0.60 mM ICD treatment for 24 h, the activities of mitochondrial respiratory chain complex enzymes I–IV were 0.035 ± 0.010, 0.438 ± 0.127, 0.170 ± 0.013, and 0.266 ± 0.026 (U/mg prot), respectively, which corresponded to 50.7%, 27.4%, 77.3%, and 73.9% of the activity of the control group, respectively (*p* < 0.001). The activity further decreased with a longer duration of treatment and higher drug concentration.

**Figure 5:**
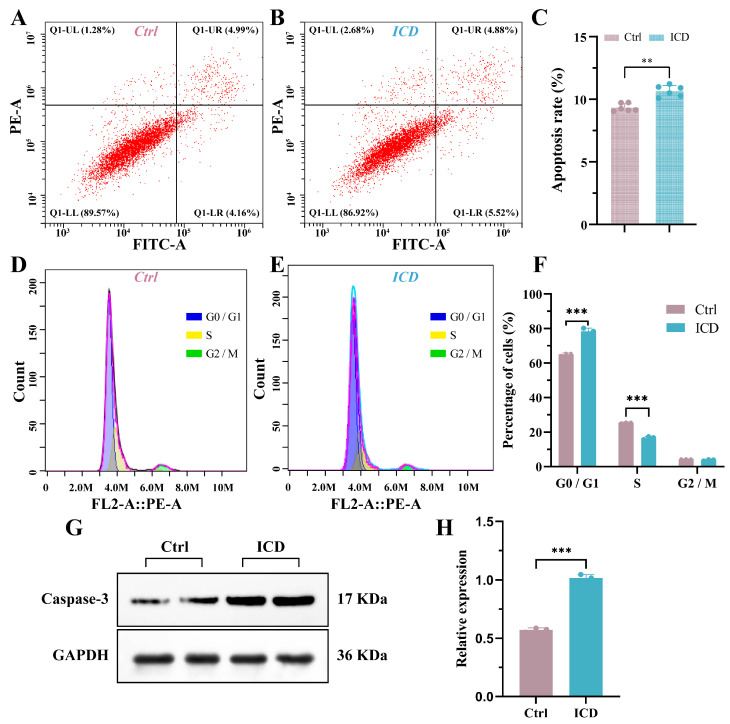
The apoptosis of Cal-27 cells increased due to ICD (**A**–**C**). Changes in cell cycle due to ICD (**D**–**F**). The expression of the Caspase-3 protein of Cal-27 was detected via Western blotting (**G**,**H**). ** *p* < 0.01; *** *p* < 0.001.

ICD reduced or even interrupted cellular energy supply and accelerated apoptosis. The apoptosis rate was about 10.57% after 24 h of action, which was different from that of the untreated group (*p* < 0.01) (Figure 5A–C). Meanwhile, the cell cycle flow showed that the percentages of G0/G1-phase and S-phase cells in the control group were 65.13% and 25.57%, respectively, but after being treated with ICD, the percentage of G0/G1-phase cells was as high as 78.4%, and the percentage of S-phase cells was reduced to 16.53% (Figure 5D–F). Additionally, Figure 5G,H showed that ICD could significantly increase the expression of Caspase 3 in Cal-27 cells. All these results indicate that ICD could promote the apoptosis of Cal-27.

## 3. Discussion

ICD has many antitumor properties and has been shown to be effective in the treatment of cancer but has not yet been used in OSCC. In this study, ICD significantly inhibited the proliferation of oral tongue squamous cells (Cal-27) in a time- and concentration-dependent manner, showing an IC50 of 0.61 mM (Figure 1B). It causes little damage to normal cells (HGFs). The therapeutic effects of ICD on OSCC were preliminarily demonstrated. To determine the underlying mechanism, a series of experiments on ROS, mitochondrial membrane potential changes, ATP content, and mitochondrial respiratory chain complex enzyme activity was conducted. ICD may cause mitochondrial dysfunction in OSCC cells, disrupting cellular energy supply and inducing mitochondria-mediated apoptosis.

Previously, ICD was found to have good anticancer activity, and further verification revealed decreased cell invasion and migration under the action of ICD (Figure 1E–H). Moreover, the expression of MMP-9 was downregulated, which significantly reduced the migration and invasion ability of cancer cells [35]. In addition, ICD can cause the depolymerization or even collapse of the cytoskeleton. The concurrent upregulation of ARP3 might be attributed to the dissolution of the Arp2/3 complex and the increase iin its related subunit, the actin-associated protein ARP3 [36].

ICD was preliminarily found to cause the skeletal deformation and dissolution of cancer cells. Therefore, the cell phenotype and mechanical properties of cancer cells were observed at the cellular microscopic level. Additionally, biomechanical studies have shown that changes in cell mechanical properties, such as cell stiffness, cell elasticity, viscoelasticity, and membrane surface adhesion, can be considered new features to characterize cancer cells [33,37]. Parameters such as Young’s modulus and cell adhesion force were quantified, revealing that cell deformability is closely related to the degree of cell malignancy [38,39]. In the current study, the AFM experiment demonstrated that Cal-27 cells treated with ICD had increased surface roughness, lower tissue stiffness (Young’s modulus), and reduced cell adhesion, migration, and invasion. Moreover, the fine skeletal structure/unit of the cells was degraded, dispersed, and difficult to distinguish, and actin filaments were almost dissolved. Tumor cell stiffness, cell viscoelasticity, and other mechanical properties are mainly determined by the actin cytoskeleton [39]. Therefore, ICD has a significant anticancer effect on OSCC which may be attributed to cytoskeletal deformation and depolymerization.

Finally, ICD was found to reduce the activity of mitochondrial respiratory chain complexes I–IV in oral squamous cell cells, thereby inhibiting the electron transport chain and blocking the energy metabolism of cancer cells, leading to cell apoptosis (Figure 4 and Table 1). This may be related to the unique metabolic mode of tumor cells, namely aerobic glycolysis, which is adapted to a low-oxygen microenvironment [40]. Some scholars have also proposed and verified the anti-tumor effect of Warburg effect inhibitors, inhibiting the activity of metabolic enzymes involved in the glycolysis pathway of tumor cells [41]. These inhibitors decrease the production of lactic acid and increase the production of ROS and mitochondrial damage, negatively affecting the mitochondrial respiratory chain. Low electron transport efficiency in the mitochondrial respiratory chain may lead to the premature electron transfer of complex I and III to O_2_, generating ROS. In addition, dysfunction of complex IV may result in an inability to couple the electrochemical gradient to ATP production [34]. In this study, ICD mainly disrupted the energy metabolism of oral squamous cell cells using this mechanism, increasing the proportion of cells in the G1 phase (Figure 5F), and ultimately inducing programmed cell death of carcinogenic cells. Moreover, some scholars have found that mitochondrial dysfunction and reduced ATP production can lead to cytoskeletal deformation and depolymerization [42]. The interaction between mitochondria and various cytoskeletal proteins is also directly involved in the regulation of mitochondrial function [43]. In this study, ICD treatment resulted in decreased ATP content in cancer cells, ROS accumulation, blocked energy metabolism, depolymerized cytoskeleton, altered cell mechanical properties, decreased adhesion, and decreased migration and invasion ability, resulting in an obvious anticancer effect.

## 4. Materials and Methods

### 4.1. Drug Preparation

ICD was purchased from Shanghai yuanye Bio-Technology Co., Ltd. (Shanghai, China. B21570). First, 5 mM of ICD was completely dissolved in dimethyl sulfoxide (DMSO) and stored at 4 °C in the dark. The complete medium was diluted to yield solutions of ICD concentrations of 0.075 mM, 0.15 mM, 0.30 mM, 0.60 mM, 1.20 mM, and 2.40 mM. The DMSO content was always kept below 0.1% (*v*/*v*). Human Tongue Squamous Cell Carcinoma (Cal-27) and Human Gingival Fibroblast (HGF) cells were purchased from Procell Life Science & Technology Co., Ltd. (Wuhan, China. CL-0265, CL-0356). The cells were cultured in DMEM with 10% fetal bovine serum (FBS) and 1% penicillin–streptomycin liquid at 37 °C and 5% CO_2_.

### 4.2. Anticancer Activity

A CCK-8 kit (K1018, APExBIO, Houston, TX, USA) was used according to the manufacturer’s instructions to measure the anticancer activity of ICD and evaluate the appropriate concentration. After the inoculated cells were completely attached to the wall, media containing different concentrations of drugs were added as experimental groups, and a medium containing the same proportion of DMSO without ICD was used as the control group. The cells were cultured for 24 h, 48 h, and 72 h. Subsequently, a 10% CCK-8 solution was added and incubated for 2 h. The absorbance value was detected at 450 nm using a multifunctional enzyme label (TECAN Infinite M200 Pro, Männedorf, Italy). Finally, the half-maximum inhibitory concentration (IC50) was calculated as previously described.

### 4.3. Migration and Invasion

The invasion and migration ability of Cal-27 cells were analyzed by cell migration and invasion assays. For the invasion assay, matrix gel (BD Bioscience, Mount Kisco, NY, USA) was added to the upper chamber, whereas no matrix gel was used for the migration assay. Then, 2 × 10^5^ cells were resuspended in 100 μL of medium without FBS, while the medium containing 10% FBS was added to the lower chamber and cultured for 24 h until the cells were attached to the wall. Then, 0.60 mM ICD was added to the upper and lower chambers. After incubation for 24 h, 48 h, and 72 h, respectively, the cells were fixed with 4% paraformaldehyde, stained with 2% crystal violet, photographed under a microscope, and counted.

### 4.4. Western Blotting (WB)

The expressions of cytoskeletal proteins (ARP3, ARPC3), migration protein (MMP-9), and apoptotic protein (Caspase3) were detected by WB. Cal-27 cells were cultured with 0.60 mM ICD for 24 h, 48 h, and 72 h, respectively. All proteins were extracted with a RIPA buffer (Beyotime, Shanghai, China) containing a protease inhibitor (Beyotime, Shanghai, China) and a phosphatase inhibitor (Roche Diagnostics GmbH, Mannheim, Germany). The protein concentration of each group was measured using the BCA protein assay kit (BCA; Beyotime, Shanghai, China). After protein denaturation, equal amounts of proteins were separated by SDS-PAGE and transferred to a PVDF membrane (Millipore, Billerica, MA, USA). The membranes were then enclosed in 5% skim milk powder at room temperature for 2 h and incubated with primary antibodies (ARPC3, ab37916, 1:1000; ARP3, ab181164, 1:1000; MMP-9, ab283575, 1:1000. Abcam; Caspase3, PA5-122288, 1:1000) at 4 °C overnight. The membranes were washed 3 times with 0.1% Tween-20 (TBST) and incubated with the appropriate HRP-chelated secondary antibodies (1:5000, Abcam, Cambridge, UK) at room temperature for 1 h. Bio-Rad Image Lab software (Version 3.0) was used to conduct an exposure analysis after ECL was added.

### 4.5. Cytoskeleton Staining

Ghost pen cyclic peptide and DAPI were used for cytoskeletal staining to observe the cytoskeletal changes induced by ICD. The cells were cultured with 0.60 mM ICD in a glass-bottom dish for 24 h, 48 h, and 72 h. Subsequently, the cells were fixed with 4% paraformaldehyde at room temperature, treated with 0.1% Triton X-100 (Solarbio, Beijing, China) for 3 min, and sealed with 1% BSA (Solarbio, Beijing, China) and 4% skim milk powder for 30 min. Cytoskeleton staining was performed with 30 µg/mL of FITC-phalloidin (Beyotime, Shanghai, China), and incubated for 60 min in the absence of light. In addition, 0.1 mg/mL of the nucleic acid dye DAPI (Beyotime, Shanghai, China) was used for nuclear re-staining at room temperature (staining for 15 min), and the stained glass-bottom dishes were observed under a confocal microscope (Carl Zeiss, Oberkochen, Germany).

### 4.6. Cell Microstructure Morphology and Mechanical Properties Analyses

Cal-27 cells were cultured with 0.60 mM ICD in a 35 cm^2^ Petri dish for 24 h and washed with PBS. Fresh medium was then added to the Petri dish. Subsequently, atomic force microscopy (AFM) (JPK NanoWizard III BioScience, Bruker, Karlsruhe, Germany) was used to analyze the mechanical properties of the cells at different drug concentrations. Images of cell microstructure morphology were acquired using the Pointprobe series of silicon AFM probes with a force constant of 0.1 N/m (Nanoworld Reflective Coatings, Yaphank, NY, USA). Fifteen random sites were selected for each sample, and each site was measured ten times. The improved Hertzian contact model was used to analyze force–distance curves, and the Young’s modulus value and roughness of cells under different drug concentrations were calculated and analyzed.

### 4.7. Cell Mitochondrial Function Analysis

Each well was seeded with 1×10^5^ Cal-27 cells. After 24 h of incubation, different concentrations of ICD were added (0, 0.15, 0.30, 0.60, and 1.20 mM) for 24 h, 48 h, and 72 h, respectively. After the cells were harvested, the levels and activities of the MMP (M8650, Solarbio, Beijing, China), ATP (BC0300, Solarbio, Beijing, China), ROS (CA1410, Solarbio, Beijing, China), and mitochondrial complex enzymes I–IV (BC0630; BC0950; BC3245; BC0945, Solarbio, Beijing, China) were detected according to the manufacturer’s instructions. The changes in the MMP in each group were detected with a 490 nm excitation wave and a 530 nm irradiation wave. The ATP content of each group was detected at a 340 nm wavelength. ROS levels were measured at a 488 nm excitation wavelength and a 525 nm emission wavelength. In addition, the cellular ROS content was calculated using flow cytometry (CytoFlex, Indianapolis, IN, USA) after 24 h, 48 h, and 72 h of treatment with 0.60 mM ICD, respectively. The detection wavelength of complex enzyme I was 600 nm, while that of complex enzyme II was 600 nm, that of complex enzyme III was 550 nm, and that of complex enzyme IV was 550 nm. The activity of the respiratory electron transport chain complex enzymes I–IV was detected in each group.

### 4.8. Cell cycle and Apoptosis Assays

After cell adherence, the cells were treated with 0.60 mM ICD for 24 h. The Annexin V-FITC/PI Apoptosis Detection Kit (E-CK-A211, Elabscience, Wuhan, China) and Cell Cycle Assay Kit (E-CK-A351, Elabscience, Wuhan, China) were used for the assay.

### 4.9. Statistical Analysis

All results were expressed as mean ± SD values, and each test was repeated at least three times. A data analysis was performed with the statistical software SPSS for Windows version 20 (IBM Inc., Chicago, IL, USA). AFM data were analyzed using JPK data processing software (version 7.0.97). The flow cytometry data analysis was performed with Treestar FlowJo (Version 10.8.1). Statistical differences were analyzed via a one-way analysis of variance (ANOVA), and statistically significant differences were considered when *p* < 0.05 (* *p* < 0.05, ** *p* < 0.01, *** *p* < 0.001, and **** *p* < 0.0001).

## 5. Conclusions

At specific concentrations, ICD can effectively inhibit the growth of oral squamous cells and destroy their cytoskeletal structure without having a serious fatal effect on normal cells. It also can induce mitochondrial dysfunction and energy metabolism disorder of cancer cells, and finally accelerate cell apoptosis. It showed good anticancer activity against OSCC, showing excellent anticancer activity against OSCC.

## Figures and Tables

**Table 1:** Respiratory electron transport chain complex enzyme I–IV activities.

Complex Enzyme (U/mg prot)	Time (h)	Ctrl	ICD (mM)				*p* Value	
			0.15	0.30	0.60	1.20		
I	24	0.069 ± 0.010	0.055 ± 0.008	0.036 ± 0.008	0.035 ± 0.010 ***	0.027 ± 0.009	0.001	0.0266 ^a^
	48	0.074 ± 0.007	0.045 ± 0.007	0.032 ± 0.014	0.022 ± 0.005 ****	0.019 ± 0.008	<0.0001	
	72	0.074 ± 0.009	0.036 ± 0.006	0.022 ± 0.008	0.016 ± 0.008 ****	0.012 ± 0.008	<0.0001	
Ⅱ	24	1.599 ± 0.201	1.167 ± 0.092	0.493 ± 0.125	0.438 ± 0.127 ****	0.367 ± 0.181	<0.0001	0.0427 ^a^
	48	1.624 ± 0.108	0.460 ± 0.100	0.427 ± 0.178	0.355 ± 0.142 ****	1.631 ± 0.154	<0.0001	
	72	0.336 ± 0.022	0.309 ± 0.040	0.292 ± 0.032	0.207 ± 0.021 ***	0.180 ± 0.025	0.0002	
Ⅲ	24	0.220 ± 0.013	0.197 ± 0.012	0.176 ± 0.011	0.170 ± 0.013 ****	0.140 ± 0.015	<0.0001	<0.0001 ^a^
	48	0.305 ± 0.039	0.174 ± 0.012	0.171 ± 0.012	0.167 ± 0.014 ****	0.146 ± 0.013	<0.0001	
	72	0.274 ± 0.010	0.145 ± 0.012	0.119 ± 0.012	0.099 ± 0.013 ****	0.067 ± 0.012	<0.0001	
Ⅳ	24	0.360 ± 0.026	0.307 ± 0.026	0.285 ± 0.027	0.266 ± 0.026 ***	0.229 ± 0.026	0.001	0.0266 ^a^
	48	0.373 ± 0.020	0.268 ± 0.026	0.255 ± 0.025	0.228 ± 0.028 ****	0.160 ± 0.026	<0.0001	
	72	0.378 ± 0.030	0.208 ± 0.027	0.174 ± 0.026	0.157 ± 0.026 ****	0.123 ± 0.027	<0.0001	

*** *p* < 0.001, **** *p* < 0.0001 vs. ctrl group. ^a^ The *p* value indicates the contrast differences between the 24 h and 72 h effects of 0.60 mM ICD.

## Data Availability

The datasets used and analyzed during the current study are available from the corresponding author upon reasonable request.

## Abbreviations

ICD: isocorydine; OSCC, oral squamous cell carcinoma; MMP-9, matrix metalloproteinase-9; AFM, atomic force microscope; MMP, mitochondrial membrane potential; ROS, reactive oxygen species.
